# Hyaluronan and cardiac regeneration

**DOI:** 10.1186/s12929-014-0100-4

**Published:** 2014-10-30

**Authors:** Francesca Bonafè, Marco Govoni, Emanuele Giordano, Claudio Marcello Caldarera, Carlo Guarnieri, Claudio Muscari

**Affiliations:** Department of Biomedical and Neuromotor Sciences (DIBINEM), University of Bologna, Via Irnerio, 48, Bologna, 40126 Italy; BioEngLab, Health Science and Technology, Interdepartmental Center for Industrial Research (HST-CIRI), University of Bologna, Ozzano Emilia, Italy; Laboratory of Cellular and Molecular Engineering "Silvio Cavalcanti", DEI, University of Bologna, Cesena, Italy; National Institute for Cardiovascular Research (INRC), Bologna, Italy

**Keywords:** Hyaluronan, Myocardial infarction, Cardiac regeneration, Adult stem cells

## Abstract

Hyaluronan (HA) is abundantly expressed in several human tissues and a variety of roles for HA has been highlighted. Particularly relevant for tissue repair, HA is actively produced during tissue injury, as widely evidenced in wound healing investigations. In the heart HA is involved in physiological functions, such as cardiac development during embryogenesis, and in pathological conditions including atherosclerosis and myocardial infarction. Moreover, owing to its relevant biological properties, HA has been widely used as a biomaterial for heart regeneration after a myocardial infarction. Indeed, HA and its derivatives are biodegradable and biocompatible, promote faster healing of injured tissues, and support cells in relevant processes including survival, proliferation, and differentiation. Injectable HA-based therapies for cardiovascular disease are gaining growing attention because of the benefits obtained in preclinical models of myocardial infarction. HA-based hydrogels, especially as a vehicle for stem cells, have been demonstrated to improve the process of cardiac repair by stimulating angiogenesis, reducing inflammation, and supporting local and grafted cells in their reparative functions. Solid-state HA-based scaffolds have been also investigated to produce constructs hosting mesenchymal stem cells or endothelial progenitor cells to be transplanted onto the infarcted surface of the heart. Finally, applying an *ex-vivo* mechanical stretching, stem cells grown in HA-based 3D scaffolds can further increase extracellular matrix production and proneness to differentiate into muscle phenotypes, thus suggesting a potential strategy to create a suitable engineered myocardial tissue for cardiac regeneration.

**Electronic supplementary material:**

The online version of this article (doi:10.1186/s12929-014-0100-4) contains supplementary material, which is available to authorized users.

## Introduction

Hyaluronan (or hyaluronic acid, HA) is a naturally occurring polysaccharide widely distributed from lower organisms, such as bacteria [1],[2], to complex eucariotes [3]. Together with collagen, HA is one of the most abundant component of the extracellular matrix (ECM). It is a non-sulfated, high molecular-weight glycosaminoglycan composed of repeating polymeric glucuronic acid and *N*-acetyl-glucosamine disaccharides conjugated by a glucuronidic β(1→3) bond and hexosaminidic β(1→4) linkages [4]. The polymeric structure of HA contains up to 25,000 disaccharide repeats in length and reaches a molecular weight of ~4,000 kDa. In cells, HA is produced by membrane-bound synthases (HAS-1 [5], HAS-2 [6], HAS-3 [7]) at the inner surface of plasma membrane and the chains are released in the ECM through pore-like structures.

The hydrolysis of the linkage between *N*-acetyl-glucosamine and glucuronic acid residues, thus HA degradation, is driven by hyaluronidases (hyaluronidases 1-5 and HYALP1), that also hydrolyze the glycosidic bonds of chondroitin and dermatan sulfates [8].

HA is present in ECM both in a soluble form and covalently bond to a variety of proteins such as proteoglycans (brevican, neurocan, versican) [9] and SHAP (serum-derived hyaluronan-associated protein) [10], often referred to as hyaladherins. Moreover, HA forms reversible linkages with water giving a specific contribution to lubrication and strength in compression in joints and soft tissues [11].

HA is also the ligand of several membrane receptors activating intracellular signaling cascades. Among them are CD44, RHAMM (receptor for hyaluronan-mediated motility expressed protein), LYVE-1 (lymphatic vessel endothelial hyaluronan receptor-1), HARE (hyaluronan receptor for endocytosis) [12], and Toll-like receptors [13]. CD44 is the major cell surface HA receptor, although it can bind also other ECM proteins, growth factors, and cytokines [14]. Most cells express CD44, including fibroblasts, smooth muscle cells, epithelial cells, and immune cells such as neutrophils, macrophages, and lymphocytes. HA-CD44 interactions play an essential role by modulating cellular growth, development, adhesion, and migration activities. Mainly through Toll-like receptors, fragmented HA is also involved in the regulation of inflammation and in immunological processes [15].

**Figure 1:**
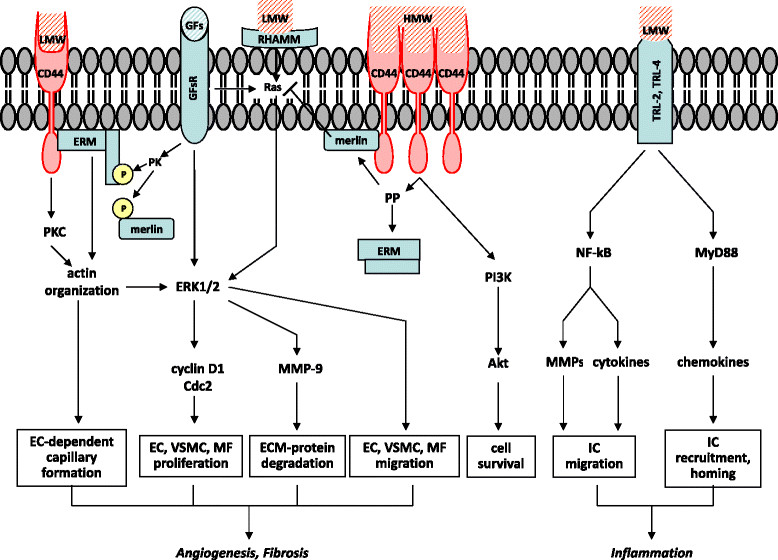
**Schematic model showing the possible receptor-mediated signal transduction pathways through which HA of different molecular weights can modulate cell functions.** High molecular weight-HA (HMW) and low molecular weight-HA (LMW) differently modulate receptor-mediated cell functions. Endothelial cells (ECs) and vascular smooth muscle cells (VSMCs) interacting with LMW can promote angiogenesis via CD44 and RHAMM transduction signaling leading to increased cell migration, cell proliferation, and tube formation. Inflammatory cells (ICs), such as monocytes/macrophages and dendritic cells, are stimulated by LMW through Toll-like receptor-2 and Toll-like receptor-4 (TLR-2, TLR-4) that promote cell migration, recruitment, and homing in damaged tissues. LMW, through CD44 and growth factors receptors (GFsR), can also induce fibroblasts and myofibroblasts (MFs) to proliferate and generate a fibrotic scar. ERM (ezrin, radixin, and moesin) protein family/merlin system seems to be largely involved in the interplay between LMW and GFs. Specifically, a non-yet identified GF-stimulated protein kinase (PK) phosphorylates ERM and promotes its migration from the cytoplasm to CD44 with consequent re-arrangement of cytoskeletal proteins that leads to cyclin D1 overexpression and increased cell proliferation. HMW exerts opposite effects, due to its antiangiogenic and antifibrotic action, at least through the activation of a CD44-dependent protein phosphatase (PP). It has been hypothesized that HMW can induce the formation of HA-receptor clusters whose signal transduction pathways are different from those stimulated by single and separated HA-receptors interacting with LMW. HMW is also involved in cell survival through the stimulation of PI3K/Akt downstream of CD44.

In humans, HA is abundantly expressed in several tissues and its different roles have been extensively reviewed, with special emphasis about those related to angiogenesis [16] and cancer [17]. HA is actively produced upon tissue injury and is significantly involved in tissue repair [18],[19]. Under these conditions, HA is more polydisperse, fragmented, with a preponderance of lower-molecular-mass forms. Low-sized fragments of HA (LMW, 100-500 kDa), but not the native high-molecular-mass HA molecules (HMW, ~4,000 kDa), stimulate inflammatory cells [20],[21]. Monocytes, macrophages, and dendritic cells migrate and home in damaged tissues thanks to the LMW stimulation of Toll-like receptors and the induction of cytokines and chemokines. These receptor-activated signaling cascades involve the nuclear factor kappa-light-chain-enhancer of activated B cells (NF-kB)/matrix metalloproteinases (MMPs) system and myeloid differentiation primary response gene (MyD88) [22] (Figure 1). LMW can also induce fibroblasts and myofibroblasts to proliferate and generate a fibrotic scar via a synergic CD44 and growth factors receptor (GFsR) -mediated signal transduction. Specifically, the ERM (ezrin, radixin and moesin) protein family/merlin system has been described to be responsible of increased cell proliferation through the organization of actin and other cytoskeletal proteins that, in turn, activate extracellular signal-regulated protein kinases 1 and 2 (ERK1/2) leading to the enhanced expression of cell cycle proteins [23].

LMW can also be considered as proangiogenic factors [24]. Indeed, vascular endothelial cells (ECs) and vascular smooth muscle cells (VSMCs) express both CD44 and RHAMM receptors and can interact with LMW stimulating both Ras/Raf/ERK1/2 and protein kinase C (PKC) pathways, then promoting cell migration, proliferation, and vessel formation [25] (Figure 1). Native HA exerts opposite effects because of its antiangiogenic properties and the block of the proinflammatory effects of LMW [26]. It has been suggested that HMW can cluster HA receptors whose intracellular signaling is different from that induced by LMW [27]. Native HA presumably activates a protein phosphatase (PP) that dephosphorylates ERM and merlin, the latter inhibiting the Ras/ERK1/2 pathway [23]. Cell survival can also be improved by native HA, e. g. through phosphoinositide 3-kinase (PI3K)/Akt stimulation downstream of CD44 [27] (Figure 1).

The molecular size of HA influences its activity in neoplastic cells. For example, only 6- to 40-mers, but not native HA, can induce CD44 cleavage [28], promote tumor cell motility in a CD44-dependent manner [29], or activate MMPs [30].

HA is abundant in the heart where it is involved in physiological functions, such as cardiac development during embryogenesis [31], and in pathological conditions including atherosclerosis [32] and myocardial infarction (MI) [33]. In the infarcted region a significant inflammatory reaction increases CD44 expression in infiltrating leukocytes, wound myofibroblasts, and vascular cells [34]. It is known that cardiac healing after a MI is paralleled by connective tissue replacement resulting in a scar. During this process an early increase of HA can be detected in the injured tissue [33]. The amount of water also gradually increased and correlated with HA accumulation. After about one month the scar was well formed and only six months later the HA content began to decrease [35]. Although these changes serve at reducing the risk of wall rupture, they usually predispose to pathological left ventricle (LV) remodeling and heart failure [36].

## Review

### Chemically modified HA for tissue engineering applications

Due to the poor mechanical properties of native HA, the clinical use of the unmodified molecule is generally limited to viscosurgery applications. Thus, several approaches are followed to improve the structural features of HA by providing chemical modifications to the HA molecule [37].

The most common structural changes in HA derive from crosslinkings performed under either acidic, neutral or alkaline conditions [38]. HA autocrosslinking occurs in the absence of potentially toxic crosslinkers and produces hydrogels through quite simple reactions. Despite the electrostatic repulsion due to its negative groups, HA can autoaggregate via hydrophobic interactions and/or hydrogen bonds between acetamido and carboxylate groups [39]. These non-covalent interactions are rather weak, so the aggregate easily forms and dissociates, depending on temperature and other environmental conditions.

Besides autocrosslinkable HA, other HA hydrogels employed in tissue engineering have been produced through added crosslinkers. More stable crosslinked HA can be obtained by means of S-S bridges, using HA-benzoyl cystein derivatives [40]. Adipic dihydrazide has also been frequently conjugated to HA through its carboxyl group to further link amine groups and peptides [41]. Water insoluble gels have been produced by crosslinking HA with glutaraldehyde (GTA), likely through hemiacetal linkages [42]. The crosslinking of the hydroxyl groups of HA using divinylsulfone provides resistant gels (hylans) through a very fast reaction in alkaline solution [43]. HA crosslinking with carbodiimides starts instead through a first reaction with carboxyl groups to form anhydrides that then interact with the hydroxyl groups. Specifically, 1-ethyl-3-(3-dimethyl aminopropyl)carbodiimide (EDC) has been widely used for this purpose, giving either biocompatible scaffolds of crosslinked HA [44] or HA conjugated with collagen or other glycosaminoglycans [45],[46]. Crosslinked HA-based scaffolds suitable for further molecular modifications have been produced with polyfunctional epoxides forming ester and ether bonds [47].

Differently from the aforementioned pre-formed chemically modified HA biomaterials, some HA-derivatives show to be crosslinked *in situ.* Such materials are in a cooled liquid form during their injection into soft tissues, hence reducing the damage due to the filling maneuver, while a rapid gelation is subsequently induced by the body temperature [48]. Light-based systems have been also described to produce photopolymerizable HA [49].

Other HA conjugates have been employed for tissue engineering applications. For example, different degrees of esterification of the HA carboxylic groups with several types of alcohols have allowed the production of both woven and non-woven meshed scaffolds with a variety of degradation times and strength resistance [50]. When HA is oxidized to form a dialdehyde, it can react with amino groups giving rise to Schiff bases, as described for HA-chitosan conjugates [51].

**Figure 2:**
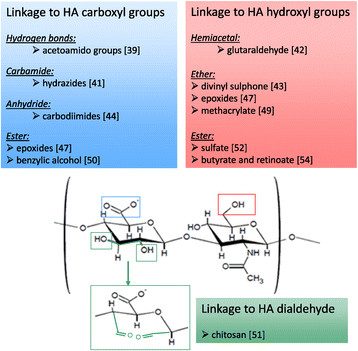
**HA structure and sites of modification.** The disaccharide repeat units of HA are shown with the primary sites for chemical modifications generally used for tissue engineering applications. The hydroxyl groups of HA form covalent linkages such as ether, esters, and hemiacetal bonds. The carboxyl groups can form ether, anhydride, and carbamide bonds, while Schiff bases can originate from HA dialdehyde reacting with amino groups. Some HA adducts commonly employed in regenerative medicine are also shown.

Finally, new drugs can be produced through different functionalizations of the HA side groups. One of the earliest HA derivatives with pharmacological properties is a HA sulfate ester showing heparin-like anticoagulant activity and high resistance to hyaluronidase [52]. Another example is a superoxide-dismutase HA-conjugate exerting anti-inflammatory activity through its antioxidant activity [53]. Butyrate and retinoate have also been esterified with HA hydroxyl groups to provide pro-differentiating properties towards grafted stem cells [54] (Figure 2).

Despite the above-mentioned modifications, HA-based materials maintain most of the biological properties of native HA, useful for tissue regeneration. Scaffolds in form of HA-hydrogels, -sponges, or -meshes [55] usually:

- are biodegradable, biocompatible, and bioresorbable [56];
- improve functions such as lubrication, cell differentiation, and cell growth [4];
- have potential to provide faster healing and repair of chronic wounds [57];
- exhibit low non-specific adsorption of proteins [9],[10] and specific with cell receptors [12] to enhance tissue growth and repair.

### Treatment of the infarcted heart with HA-based biomaterials

#### HA-based hydrogels without cellular components

Several studies show that injectable HA-based hydrogels are rather effective in regenerating the damaged cardiac tissue after a MI. Yoon et al. induced a MI in rats via circumflex artery ligation and an acrylated HA hydrogel, obtained using a polyethylenglycol-thiol (PEG-SH_4_) cross-linker, was injected into the epicardium of the infarcted region [58]. A significant decrease in both infarcted area and apoptotic index followed the treatment with the hydrogel, while the number of arterioles and capillaries increased. Parameters of heart function, such as ejection fraction, dP/dt max, and dP/dt min also improved in treated animals. When 50 kDa, 130 kDa, and 170 kDa HA-based hydrogels were applied to the infarcted area in a sub-acute model of MI, the lowest sized hydrogel provided the most significant myocardial regeneration and functional recovery of the heart. Conversely, independently of the HA size, cardiac regeneration did not occur in a chronic model of MI, reflecting that the injection timing of the therapeutic agents is also a major determinant in the process of cardiac repair [59]. Thus two major factors, i.e. molecular weight of HA and progression of MI, are responsible for HA-dependent regeneration in MI.

HA-based hydrogels with different degradation times were produced by Tous et al. [60]. Two hydrolytically degrading 66 kDa HA hydrogels (~3 and 10 weeks, respectively), with low and high degree of crosslinking (low: ~7 kPa; high: ~35-40 kPa), and two more stable HA hydrogels with similar low and high initial mechanics, were evaluated in an ovine model of MI. The most stable hydrogels were obtained using metacrylate to form crosslinkable macromers. LV wall thickness increased in the infarcted area injected with all hydrogels compared to the untreated controls and the most stable HA maintained the wall thickness longer than the hydrolytically degrading hydrogels. Moreover, while both HA with high mechanics initially reduced LV volumes, only the most stable hydrogel with high mechanics was more effective after a longer period, implying that wall stabilization requires a certain time for volume maintenance. Consistently with the regeneration effects induced by HA, the injection of these HA-hydrogels resulted in better cardiac output values than those observed in the untreated infarcted hearts. A computational study investigating the mechanical properties in myocardium after HA hydrogel injection [61] argued that this treatment can enhance the stiffness of the myocardium/hydrogel composite region in an anisotropic manner and increase the modulus in the longitudinal direction compared to control myocardium. In these simulations, the overall increased stiffness, in combination with the augmented volume due to hydrogel injection, partially reduced the global fiber stress.

Taken together, these results show that a relevant mechanism underlying the benefits induced by HA-based hydrogels is attributable to their bulking (thickening) and stabilizing effects on the infarcted region. Fiber stress in the injured myocardium can be thoroughly decreased depending on hydrogel stiffness, that is regulated by its own mechanical properties and degradation behavior. Furthermore, these findings support the notion that small HA fragments exert opposite effects than longer HA molecules by promoting angiogenesis. Interestingly, the addition of VEGF to 50 kDa HA fragments did not further increase vessel density in the infarcted area, underlining the great efficacy of low molecular weight HA in stimulating the process of vascularization.

In the attempt to obtain a sustained delivery system, Purcell et al. developed *in situ* forming HA hydrogels with degradable crosslinks to regulate the release of both recombinant stromal cell-derived factor-1α (rSDF-1α) and HA fragments to the injured myocardium [62]. This strategy was adopted in order to enhance the homing of circulating bone marrow cells in the myocardium following a MI. A degradable 74 kDa HA with hydroxyethylmethacrylate (HeMA) functionality was formed as a crosslinkable hydrogel upon a brief visible light exposure using a chemical initiator system. The diffusion of the encapsulated rSDF-1α was slow because of the electrostatic interactions between the negative-charged carboxylic groups of the HeMA-HA and the positive-charged basic aminoacid residues exposed on the external domains of the chemokine. The release of both rSDF-1α and crosslinked HA was sustained for over seven days and these compounds stimulated *in vitro* bone marrow cell chemotaxis through CXCR4 and CD44 receptors, respectively. Moreover, the homing of circulating marrow cells significantly increased in the infarcted region injected with the HeMA-HA hydrogel plus rSDF-1α, compared to rSDF-1α or HeMA-HA alone. The binding of HA to CD44 stimulated G-protein-dependent bone marrow cell motility through Rho-associated protein kinase (ROCK) signaling [63] and cell adhesion through integrin expression [64]. A fluorophore-conjugated SDF-1α analogue was also associated to 74 kDa HA and chemically modified with HeMA to form a hydrolytically degradable hydrogel only at body temperature [65]. In this study HA gelation was rapidly induced *in vivo* through a free-radical initiator system. HA hydrogel degradation, together with the complete release of the SDF-1α analogue, took more than one month. The mixed SDF-1α analogue/HA hydrogel injected into rat cardiac border zone significantly improved vascularity, ventricular geometry, ejection fraction, cardiac output and contractility. According to a similar strategy, the release of recombinant tissue-inhibitor-3 of matrix metalloproteinase (TIMP-3) from a degradable HA hydrogel counteracted pathological LV remodeling after experimental MI. This effect was due to a marked reduction in pro-inflammatory cytokines as a consequence of the sustained delivery of TIMP-3 in the border zone [66].

Another set of experiments carried out by Ventura et al. explored the effects of HA-mixed ester of retinoic acid and butyric acid, alone or in combination with stem cells, on cardiovascular repair and functional recovery [54],[67],[68]. All-trans retinoic acid and butyric acid were conjugated with 85 kDa HA providing mixed HA esters. Their general formula consisted of three distinct dimeric repeating units, two of them are retinoylate or butyrylated in the hydroxyl group of the N-acetyl-D-glucosamine residues, usually in position 6, whilst the third unit is non-substituted. The number of the esterified OH groups for each disaccharide unit of HA (degree of substitution, DS) ranged between 0.002 and 0.1 with retinoic acid and between 0.05 and 1.5 with butyric acid. HA-mixed esters were previously demonstrated to afford high throughput of cardiogenesis in embryonic stem cells [69]. Indeed, CD44 is abundantly expressed in the embryonic myocardium and the differentiation process is accompanied by an increased expression of CD44 [70]. Positron emission tomography (PET) showed that HA-mixed esters with DS of retinoic acid and butyric acid of 0.032 and 1.44, respectively, injected alone in the infarcted rat heart, restored cardiac [^18^ F]fluorodeoxyglucose uptake as well as increased both capillary density and recruitment of endogenous Stro-1-positive stem cells [67]. The stimulation of vascularization was likely the consequence of the enhanced gene expression and secretion of VEGF and HGF from stem cells exposed to the HA-mixed ester. Treated hearts also exhibited a decrease in the number of apoptotic cardiomyocytes, due to putative enhanced expression of *Akt* and *pim-1* survival genes. HA-mixed ester injection increased H4 histone acetylation and recruited Stro-1 positive stem cells in the infarcted myocardium, suggesting that a stimulation of the differentiation potential was also achieved through this treatment. Nuclear run-off experiments indicated that the action of HA-mixed ester was modulated at the transcriptional level, although nuclear exposure to HA-mixed ester did not affect gene transcription. Therefore, it has been hypothesized that retinoic acid and butyric acid hydrolyzed from the HA-mixed ester stimulated DNA transcription. Nevertheless, the molecular mechanism underlying HA-mixed ester-mediated responses, as well as the role of HA itself, remain to be elucidated. The HA-mixed ester was able to enhance cardiac regeneration in a more relevant manner when associated with adult stem cells, as described in the following paragraph.

#### HA-based hydrogels with a cellular component

HA hydrogels are effective in cardiac repair because they activate several mechanisms of myocardial regeneration but also for their ability to serve as a scaffold for stem cell transplantation. An example of a synergistic action between a HA-based hydrogel and mesenchymal stem cells (MSCs) was shown by the above-mentioned mixed esters of HA with butyric acid and retinoic acid [54]. MSCs isolated from bone marrow, dental pulp, and fetal membranes of term placenta were first cultured in the presence of the HA-mixed ester. Angiogenic, mitogenic, and antiapoptotic factors were upregulated under these conditions and MSCs were committed into the endothelial and cardiac cell lineages. Early cardiac genes, such as GATA-4 and Nkx-2.5, as well as sarcomeric genes such as myosin heavy chain and α-actinin, were significantly expressed in MSCs isolated from placenta and transplanted into infarcted rat hearts, where they increased capillary density and decreased scar size. Moreover, when placental MSCs were preconditioned with the HA-mixed ester, angiogenesis further increased although only a few engrafted cells stained positive for cardiomyocyte markers. Since the exposure of MSCs to a CD44 blocking antibody abrogated the HA-mixed ester-mediated MSC commitment to both cardiac and endothelial phenotypes, it is conceivable that the involvement of this receptor represents a necessary step for this process. In addition, the finding that HA decreased the yield of committed MSCs in a dose-dependent manner suggests the participation of a CD44-related uptake system for the HA-mixed ester entry into the cell.

When the same experiments were performed in the infarcted pig heart, the injection of placental MSCs pre-treated with the HA-mixed ester improved both end-systolic wall thickening and circumferential shortening of the infarct border zone, and decreased the infarct area by 40% in comparison to the untreated infarct hearts [68]. Under this condition, PET showed that both myocardial perfusion and metabolism were improved. This animal model of MI allowed to underline that the transplanted MSCs pretreated with HA-mixed ester seem to differentiate only as vascular cells but not as cardiomyocytes. By contrast, the protective and regenerative effects on surviving cardiac muscle cells were obtained through the release of paracrine factors from MSCs. Interestingly, proteomic analysis of the border zone showed a phenotypic homology with healthy cardiac tissue in the group injected with pretreated placental MSCs. Specifically, the small-leucin-rich proteoglycan lumican, which contributes to assembly collagen fibers in the fibrotic scar, was not upregulated, while mitochondrial respiratory enzymes and carbonic anhydrase-I were not downregulated in the hearts grafted with HA-mixed ester treated MSCs.

The efficacy of the transplantation of mononuclear bone marrow cells (MNCs), was investigated in MI using a HA hydrogel as a vehicle [71]. Bone marrow MNCs are a heterogeneous cell population that consists of MSCs, hematopoietic stem cells, and endothelial lineage cells, all of them showing beneficial effects on cardiac repair [72]. HA of 1,630 kDa MW dissolved at 1% in PBS formed a hydrogel that provided a favorable microenvironment for MNC adhesion, proliferation, and vascular differentiation in standard culture condition. The injection of MNCs embedded in HA hydrogel in the infarcted heart significantly reduced inflammation, cardiomyocyte apoptosis, and infarct size. HA hydrogel improved MNC retention in the injured area and promoted angiogenesis. Based on flow-cytometry analysis, 56% MNCs expressed CD44, while RHAMM expression was only 0.6%; therefore, CD44 binding with HA seemed to be responsible, at least in part, of the improved cell adhesion, survival, and differentiation. It is important to note that MNCs cultured in the presence of HA secreted more survival and pro-angiogenic factors, such as FGF-2, HGF, IGF-1, PDGFb, and SFD-1, compared to standard MNCs. This study also highlighted that cardiac genes such as α-MHC, and cTnI did not increase in MNCs after exposure to HA, suggesting that HA *per se* cannot stimulate stem/progenitor cell differentiation into the cardiac lineage.

The effect of MNCs suspension in 1% HA hydrogel on post-MI regeneration was also recently investigated in mini-pigs by the same Authors [73] and associated with a significant increase in LV ejection fraction, contractility, and neovascularization, consistent with a decrease in infarct size. Interestingly, this combined treatment was superior in improving myocardial performance than HA or MNC injection alone. Transplantation of MNCs with HA also ameliorated cell grafting and differentiation into the vascular lineage.

Besides bone marrow-derived stem cells, cardiosphere-derived cells (CDCs) have been largely investigated as promising cells for cardiac tissue regeneration [74]. In order to improve acute CDC retention in the myocardium, a HA-blood hydrogel was synthesized by mixing lysed whole blood and 16 kDa HA in a 1:1 (v/v) ratio. HA carboxyl groups were functionalized with N-hydroxysuccinimide (NHS) to yield covalent crosslinked HA succinimidyl succinate [75]. This hydrogel (gelation time ~60 s) was injected intra-myocardially, or applied epicardially, in a rat model of MI. NHS-activated carboxyl groups of HA reacted with the primary amines of both blood and myocardium and formed amide bonds, resulting in a 3D hydrogel bound to the tissue. CDCs isolated from rat hearts and cultured in HA-blood hydrogel increased their survival and rate of proliferation. When CDCs encapsulated in this hydrogel were injected in the infarcted area their retention was enhanced. Indeed, the epicardial application of the HA-blood hydrogel alone improved LV ejection fraction. The addition of autologous blood to HA provided adjunctive adhesion motifs in blood vitronectin and fibronectin, such as arginine-glycine-aspartate (RGD) sequence, activating integrin and pro-survival pathways. Lysed blood also increased the compressive module of the HA hydrogel resulting in further improvement in cardiac function [76]. Moreover, the porosity of this blood-HA hydrogel showed a high swelling ratio that allowed an enhanced exchange of electrolytes/metabolites and better cell infiltration.

CDCs isolated from human cardiac biopsies have been investigated by Marban's group [77] who employed an *in situ* polymerizable hydrogel (Hystem®*-C*™) as a vehicle [78]. Hystem®*-C*™ is a HA and porcine gelatin hydrogel that can be formulated as a liquid and forms a gel at 37°C within 20 min. This HA-based hydrogel was crosslinked using thiol-reactive poly(ethyleneglycol)diacrylate and covalently linked to collagen to improve cell adhesion. CDCs, which express multiple collagen-binding integrins (α1, α2, α3) and CD44, were found to be highly compatible with this hydrogel. In Hystem®*-C*™ CDCs acquired a spread morphology, like seen in standard culture plates, while they remained rounded and more than 50% died within one week in the base product without collagen. Additionally, the *in vitro* migratory capacity of CDCs, as well as CDC retention, resulted increased vs. HA alone when using Hystem®*-C*™ in a mouse SCID model of MI. These modifications led to amelioration in cardiac function and to a reduced degree of adverse remodeling. Thanks to the improved engraftment, CDC delivery in Hystem®*-C*™ resulted in an increased number of new cardiomyocytes and endothelial cells. Therefore, most of these effects were attributed to the improvement in cell retention and to the consequent increase in CDC paracrine activity rather than the differentiation of the grafted cells toward a cardiomyocyte phenotype. Proofs of the involvement of Hystem®*-C*™ alone in cardiac repair were provided by Abdalla et al. who demonstrated that this hydrogel was also able to induce a certain degree of neovascularization and to improve cardiac function in a rat model of MI [79].

#### HA-based patches with a cellular component

Biopolimer-based patches for repairing the infarcted heart have been manufactured using natural, semi-synthetic, or fully synthetic materials [80]. Differently from injectable hydrogels, the mechanical strength of polymeric patches ensures a superior reinforcement of the infarcted wall as well as the possibility to build *in vitro* an engineered cardiac tissue to be transplanted in the damaged myocardium. Among semi-synthetic polymers used to improve cardiac regeneration, our group explored the effects of solid-state structures of HA-based scaffolds. The fine mechanisms of the interaction of rat MSCs with a HA-based polymeric support were initially investigated to evaluate the potential clinical application of these constructs [81]. A water insoluble scaffold, HYAFF®11 resulted from the total esterification of the carboxylic groups of non-woven 200 kDa HA with benzylic alcohol [50]. The high biocompatibility and biodegradability of this semi-synthetic biomaterial afforded suitable features for cell growth and 3D tissue reconstruction, as well as for the safe transplant of the material to injured body sites [82],[83]. Specifically, the *in vitro* hydrolytic degradation of the ester bonds of HYAFF®11 is completed after about two months in artificial plasma and not later than four months in biological tissues. Non-woven HYAFF®11 showed 15μm-average fiber diameter and seeded MSCs remained viable for a long time, growing on the surface and in the innermost portions of the scaffold. Early culture showed MSCs wrapping individual fibers with regularly spaced focal contacts and fibronexus formation. Despite the diameter of the fibers was not in the nanoscale range, it is conceivable that the high number of focal contacts might regulate fundamental cell functions such as motility, proliferation, and survival by integrin-mediated cell signaling. Interestingly, the density of CD90, a putative marker of mesenchymal stemness, was unaffected after two weeks of cell culture. Moreover, a polarized cell membrane expression of CD44 was showed in correspondence to the HYAFF®11-cell contacts, suggesting that these cell regions can be particularly prone to translate the interaction of HA with its receptor into intracellular signaling. MSCs were able to steadily proliferate on HYAFF®11 and no significant cell loss or degenerative modifications were found within three weeks of cell culture. Another consequence of MSCs- HYAFF®11 interaction was the increased production of proteoglycans, including versican and decorin, whose role as repositories of growth factors and cytokines can also contribute to tissue regeneration [84].

Non-woven HYAFF®11 appeared as a suitable substrate also for human EPCs, that showed extensive adhesion and viability on such a scaffold [85]. Similarly to MSCs, EPCs easily migrated to and aggregated on the scaffold and active protein synthesis and features of endothelial differentiation were revealed showing some degree of angiogenic activity.

Since these findings indicated that non-woven HYAFF®11 could be considered as a promising cell vehicle, we tested the feasibility to graft a MSC/non-woven HYAFF®11 construct in the scar of infarcted rat hearts [86]. Two weeks after coronary ligation, a small disk of the construct was introduced into a pouch created in the ventricular wall of the infarcted area. Within two weeks most cells migrated from the grafted HYAFF®11 towards the border zone close to coronary vessels and the graft induced angiogenesis and attenuated the fibrotic process in the infarcted region. To better understand the mechanisms underlying the specific roles of MSCs and HYAFF®11 this construct was also tested in a pre-clinical pig model of MI [87]. The scaffold adopted was again the total benzyl ester of HA formulated as a semi-elastic woven mesh to support LV cyclic movements. Autologous bone marrow MSCs were cultured onto this scaffold for four weeks leading to the formation of a cardiac patch suitable for transplantation. Female pigs were subjected to a permanent left anterior descending coronary artery ligation and scar perfusion was evaluated by Contrast Enhanced Ultrasound echography eight weeks after MI. The HYAFF®11/MSC group significantly increased the ratio between the percent peak perfusion and the time to peak, reaching levels of scar perfusion comparable with those of the healthy, non-infarcted hearts. The grafted construct also reduced the infarction-related inflammation and the foreign reaction against the graft remained strictly localized around the fibers of the scaffold. Cardiac tissue positively interacted with the construct by reducing the presence of collagen and increasing the amount of proteoglycans. Moreover, the cardiomyocytes in the border-zone favorably reacted to the graft and a lower degree of cellular damage was found. Thus, the transplantation in the myocardial infarct area of autologous MSCs supported by a HA-based scaffold restored blood perfusion in the ischemic area and almost completely abolished the inflammatory process, showing that these benefits were superior to those obtained grafting the scaffold or the MSCs alone. The ability of grafted MSCs to promote angiogenesis through their paracrine activity has been described in other models of MI [88]. In synergy to MSCs, the increase in vessel density stimulated by HYAFF®11 alone could be related to its gradual fragmentation into smaller-sized HA olygosaccharides. The foreign inflammatory reaction localized around the fibers of the scaffold might have accelerated this breakdown. On the other hand, the presence of MSCs attenuated the inflammatory process reactive to MI, probably owing to the innate immunosuppressive properties of these cells. Interestingly, the native cardiac tissue positively interacted with the HYAFF®11/MSCs construct by reducing the amount of collagen and increasing the amount of proteoglycans in the ECM. A lower degree of cardiomyocyte damage was observed as well in the border zone.

Another mesh useful to graft MSCs in the infarcted myocardium was prepared with HA and silk fibroin [89]. The rationale of this study was that such blends would allow the combination of the superior mechanical properties provided by fibroin with the biological features of HA. The scaffold was prepared by freeze-drying aqueous solutions of fibroin and HA subsequently incubated in methanol to induce water insolubility and formation of microporous structures. MSCs cultured on silk fibroin/HA scaffolds showed enhanced cellular ingrowth and increased glycosaminoglycan and type-I / type-III collagen gene expression, as compared to plain silk fibroin scaffolds. CD44-blockage treatment decreased MSC growth rate of about 50% and fibronectin expression was reduced [90]. Cardiomyogenesis induced by 5'-azacytidine on MSCs, documented by GATA-4, Nkx2.5, Tnnt2, and Actc1 gene expression and upregulation of later cardiac markers such as cardiotin and connexin 43, could occur on fibroin/HA patches. Indeed, it is known that this nucleotide analogue can be usefully employed for MSC commitment into a cardiomyocyte-like phenotype before transplantation [91],[92]. CD44-blockage abolished this initial commitment toward a cardiac muscle phenotype, suggesting at least a permissive role of this receptor also in cardiac specification. Therefore, CD44 influenced proliferation, fibronectin expression, and cardiomyogenic differentiation in MSCs cultured on fibroin/HA patches. When implanted into MI rat hearts this cell-engineered construct remained intact for a long time and attenuated the local immunological responses, prevented cardiomyocytes apoptosis, stimulated the secretion of VEGF, bFGF, and HGF, and improved LV wall thickness, MSC viability, and myocardial neo-vascularization [93].

Yang et al. deepened the investigations on HA/silk fibroin-based scaffold introducing a chitosan component and evaluating its *in vitro* cardiomyogenic effects on MSCs [94]. Chitosan is an aminopolysaccharide showing chemo-attractive properties for growth factors and high capacity to impact with several compounds, as demonstrated during bone regeneration [95]. The molecular weight of HA was 15 kDa, yielding a 1:1:10 (w/w/w) chitosan/HA/fibroin ratio. Specifically, HA and chitosan were incorporated to, or co-sprayed with, fibroin to produce microparticles that were further processed by mechanical pressing and genipin cross-linking to produce hybrid cardiac patches. MTT viability assay demonstrated that MSC expansion and rate of proliferation on fibroin and fibroin-hybrid patches significantly exceeded that on traditional culture wells and the expression of specific cardiac markers significantly increased in MSCs treated with 5'-azacytidine grown on fibroin/chitosan-HA. The same Authors demonstrated that fibroin/chitosan-HA patches without a cellular component improved LV performance by reducing the inner diameter dilation and increasing LV wall thickness and fractional shortening, compared to the untreated rats [96]. Moreover, the secretion of angiogenic factors and vessel density were increased in the infarcted region of LV. Thus, it appears that the presence of chitosan in the HA/fibroin patch can exert beneficial effects on the damaged myocardium similarly to those described when the HA/fibroin scaffold was grafted with MSCs. However, the molecular mechanisms underlying the *in vitro* and *in vivo* responses mediated by the different scaffolds containing fibroin, chitosan, and HA, as well as their single contribution, remain to be elucidated.

#### Mechanical stretch of HA-based cell constructs

Several experimental evidences have demonstrated that mechanical forces can modulate intracellular signaling and gene expression and affect fundamental process such as cell growth and differentiation [97]-[99]. Studies on the effects of cyclic mechanical stress on human cardiomyocytes grown in a 3D matrix also showed that this technique promoted a significant increase in cardiomyocyte number size [100], enhanced myofibrillogenesis, and induced the alignment of collagen fiber bundles in the ECM [101].

Hybrid cardiac scaffolds with mechanical properties suitable for *in vitro* loading studies and *in vivo* implantation were also constructed from neonatal rat heart cells, fibrin, and the HA-based woven mesh HYAFF®11 [102]. Early after cell seeding, stiffness was half as high as that of native heart, whereas ultimate tensile strength, failure strain, and strain energy density significantly increased in respect to native heart. Constructs implanted subcutaneously in nude rats exhibited a high degree of cardiomyocyte differentiation and blood vessel ingrowth. Although a significant increase in construct collagen content and maintenance of stiffness was demonstrated under static culture conditions, cyclic stretch further increased collagen biosynthesis in a load-dependent manner. These findings implied the potential of woven HYAFF®11 for *in vivo* remodeling in response to biochemical and physical factors mimicking the contracting myocardium.

According to these preliminary results, we then investigated whether MSCs seeded onto the HYAFF®11 woven mesh scaffold could be addressed towards a muscle phenotype via the transfer of a controlled and highly-reproducible cyclic deformation [103]. The construct that was obtained after one week of mechanical stretch showed cells displaying multilayer organization, invading the 3D mesh of the scaffold, and expressing typical markers of muscle cells. These effects were due only to physical cell stimulation, without the need of any other chemical or genetic manipulation. Therefore, we suggest to explore the efficacy of mechanically-stimulated MSC/HA-based construct as a reliable engineered myocardial tissue for future investigations on cardiac regeneration [104].

Since not long ago, fascinating new discoveries obtained by Chopra et al. [105],[106] helped to understand biological and molecular mechanisms involved in the interaction between HA and cells, highlighting how HA is able to change the mechanical response of neonatal cardiac myocytes *in vitro* and allow their functional development on HA gels. HA matrix by itself is not adhesive for myocytes and often considered to be inert, but when adhesive ligands, such as fibronectin, collagen I, laminin, collagen IV, or cadherin are incorporated, they allow to host well-developed myocytes promoting the formation of functional sarcomeres. In fact, HA interacts with cells through its receptors CD44, RHAMM, layilin, and ICAM-1 and can also bind fibronectin and collagen VI. Thus, HA alters the integrin-dependent stiffness response of cells *in vitro* suggesting a similar alteration *in vivo*, probably modifying the response of cells that bind the ECM through integrins. It was recently confirmed that the presence of long unmodified HA polymers alters the mechanosensing signals mediated by the activated integrin and induces the acquisition of a phenotype that cannot be attained under the same mechanical conditions by integrin engagement alone [105]. Indeed, these results provide a rationale to produce a new class of soft hybrid scaffolds useful in cardiac tissue engineering and regenerative medicine.

## Conclusions

**Figure 3:**
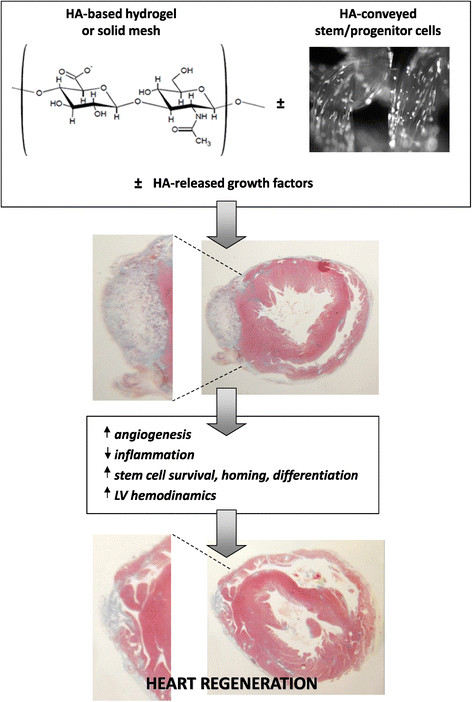
**Potential effects of HA-based scaffolds and conveyed stem/progenitor cells on cardiac regeneration after MI.** HA-based biomaterial can be injected as a hydrogel or grafted as a solid mesh providing improvements in left ventricular (LV) structure and functions. Delivered stem/progenitor cells and/or released growth factors can further increase regeneration efficacy by promoting cell survival, reducing inflammatory reaction, increasing neoangiogenesis, and favoring resident or transplanted cell differentiation.

Regeneration of the infarcted region of the myocardium represents a challenge to reduce the risk of contractile failure that usually follows the pathological remodeling of the scarred area. Tissue engineering approaches employing biomaterials and/or stem cells represent the hope to heal a damaged heart after MI. HA can be used in its native form as a vehicle for repairing cells, but more useful derivatives are preferred due to their lower degradability and increased mechanical strength. When grafted in the infarcted heart, HA-based scaffolds improved myocardial structure and functional parameters by promoting cell survival, reducing inflammatory reaction, increasing neovascularization, and favoring cardiovascular commitment of resident or transplanted stem cells (Figure 3), although to a lower extent. HA-based hydrogels show the advantage to be injected by transcatheter technologies, while the solid-state counterpart usually needs open-heart surgery. However, care must be taken in modulating hydrogel density to avoid laceration of myocardial tissue network in the injected area, especially when cells are simultaneously delivered. On the other hand, HA in both form of woven or non-woven mesh can be applied as a patch on the myocardial surface reinforcing the cardiac scar and providing conveyed stem cells for a sustained process of repair. Adult stem cells transplanted with HA-based scaffolds increased their viability and improved retention in the grafted region and a CD44-dependent mechanism has been often invoked like possible responsible for this benefits. Stem cell differentiation into cardiomyocyte phenotype was also advantaged by the presence of HA-based scaffolds, especially when conjugated with other factors which offer synergistic effects for cardiac gene expression.

Clinical trials using HA-based materials alone or with cellular components have been performed in the attempt to obtain a regenerative action mainly as wound healing and osteo-articular repair. Indeed, most of these studies underlined the benefits that the HA derivatives, either as hydrogel or solid mesh, exerted in human diseases such as venous ulcers [107],[108], decubitus ulcers [109], oral mucosal lesions [110], surgical ablation of nevi/cutaneous tumors and cicatritial outcomes [111], talar dome and knee osteochondral lesions [112],[113], and cranial bone regeneration [114]. So far, no clinical trial started to verify whether such biomaterial can be useful also to repair the infarcted myocardium. However, the cumulative literature here reviewed suggests that HA-based compounds represent a safe biomaterial for cardiac tissue engineering, providing a suitable environment able to improve the survival and the functions of resident and grafted cells.

## Authors' contributions

All authors designed the concept and collected information. FB elaborated the figures. CM wrote the manuscript and MG, EG particularly contributed to the section on the effects of mechanical stretch. All authors read, revised critically, and approved the final manuscript.

## Authors’ original submitted files for images

Below are the links to the authors’ original submitted files for images. Authors’ original file for figure 1 Authors’ original file for figure 2 Authors’ original file for figure 3
