# Supplementary figures and images for: Hyaluronan and cardiac regeneration
Source: J Biomed Sci. 2014 Oct 30;21:100. doi: 10.1186/s12929-014-0100-4 (PMC4226915; doi:10.1186/s12929-014-0100-4)

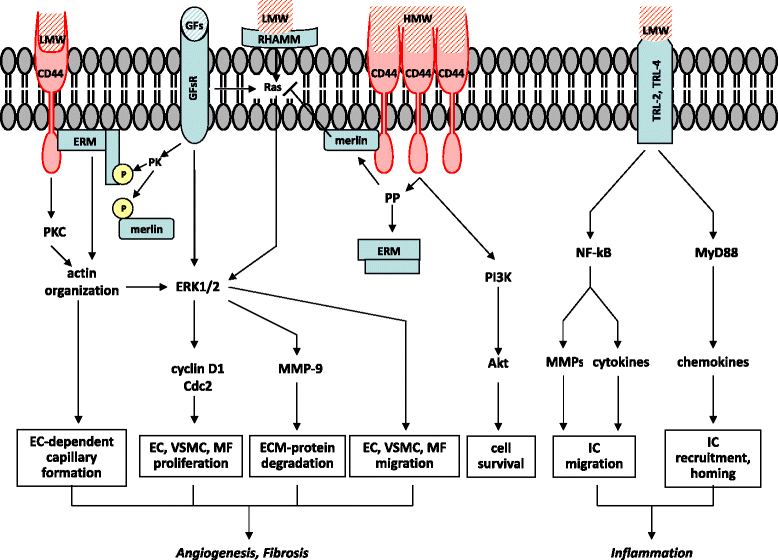

Supplement: Supplementary file 1 — Authors’ original file for figure 1 [file 12929_2014_100_MOESM1_ESM.gif]

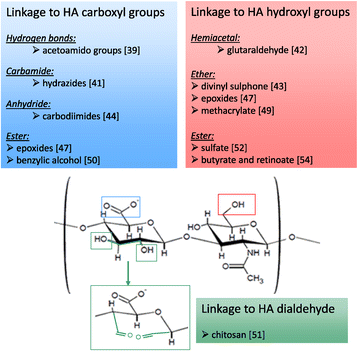

Supplement: Supplementary file 2 — Authors’ original file for figure 2 [file 12929_2014_100_MOESM2_ESM.gif]

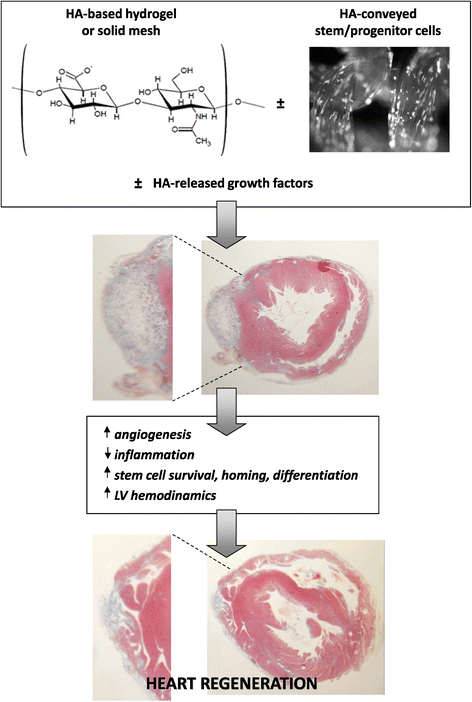

Supplement: Supplementary file 3 — Authors’ original file for figure 3 [file 12929_2014_100_MOESM3_ESM.gif]
